# CD28 and chemokine receptors: Signalling amplifiers at the immunological synapse

**DOI:** 10.3389/fimmu.2022.938004

**Published:** 2022-08-02

**Authors:** Barbara Molon, Cristina Liboni, Antonella Viola

**Affiliations:** ^1^ Pediatric Research Institute “Città della Speranza”, Corso Stati Uniti, Padova, Italy; ^2^ Department of Biomedical Sciences, University of Padova, Padova, Italy

**Keywords:** immune synapse, Chemokine receptors, CD28, costimulation, lipid raft

## Abstract

T cells are master regulators of the immune response tuning, among others, B cells, macrophages and NK cells. To exert their functions requiring high sensibility and specificity, T cells need to integrate different stimuli from the surrounding microenvironment. A finely tuned signalling compartmentalization orchestrated in dynamic platforms is an essential requirement for the proper and efficient response of these cells to distinct triggers. During years, several studies have depicted the pivotal role of the cytoskeleton and lipid microdomains in controlling signalling compartmentalization during T cell activation and functions. Here, we discuss mechanisms responsible for signalling amplification and compartmentalization in T cell activation, focusing on the role of CD28, chemokine receptors and the actin cytoskeleton. We also take into account the detrimental effect of mutations carried by distinct signalling proteins giving rise to syndromes characterized by defects in T cell functionality.

## Signalling compartmentalization: surrounding molecules to integrate and amplify signals

Cells must be able to sense, decode and integrate a plethora of environmental stimuli. For many years, an outstanding question for cell biologists was how different signalling cascades, exploiting common intracellular effectors, could trigger distinct cellular responses. It is now clear that to allow the proper progress of specific cellular responses, signalling effectors must be locally confined in space and time, a concept referred as signalling compartmentalization. The tight control of the location, duration and frequency of signalling molecules indeed contributed to the relevant functional specificity that enables receptors to encode distinct cellular responses.

Protein compartmentalization is integral to achieving effective and controlled T cell responses, which are drivers of the adaptive branch of the immune system (1, 2). Over the last years, multiple studies have shed light on both the mechanisms by which signals are compartmentalized in T cells and the physiological role played by such compartmentalization (3). Both lymphocyte migration and activation indeed rely on the selective and transient segregation of signalling molecules and membrane receptors that localized in specific cell locations with different kinetics.

The dynamic molecular compartmentation of signalling players in T cells is ensured by the interplay between the plasma membrane (PM), cytoskeleton networks, and intracellular organelles.

Collectively, such events lead to the establishment of a morphological and molecular asymmetry know as lymphocyte polarization which is crucial for T cell migration and activation.

**Figure 1:**
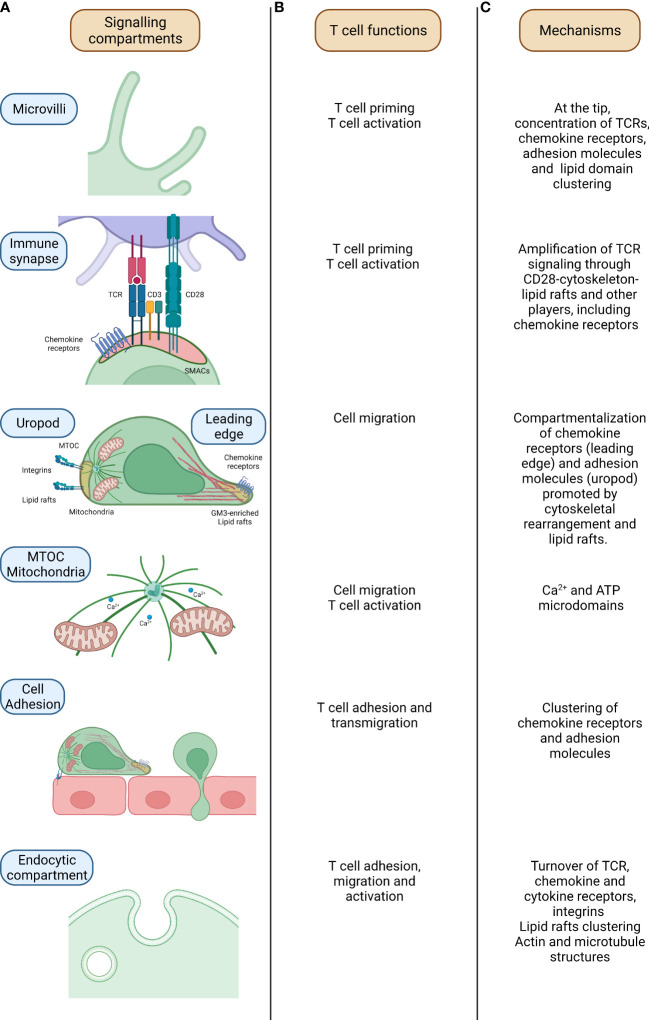
Mechanisms of signalling compartmentalization in T cells. From the left, relevant compartments which regulate signalling compartmentalization in T cells **(A)**, related T cell activities **(B)** and mechanisms underpinning signalling compartmentalization at these sites are outlined **(C)**. In microvilli, parallel actin filaments allow the sustainment of the structure which assure the concentration of proteins and molecules and signalling compartmentalization in naïve T cells. In the immune synapse (IS), where the formation of the couple between the T cell and the APC is assured by the specific recognition of the Ag recognized on the MHCII molecules by the TCR, the binding of the two cells is further sustained by the CD3 and co-stimulatory molecules (CD28/B7-1/CD80-B7-2/CD86). Here, the compartmentalization of the signalling is mediated by the concerted action of cytoskeletal components, lipid rafts and endocytic compartment. During T cell migration, the T cell acquires an intrinsic polarity mandatory for the definition of a leading edge and a rear pole (uropod). The differential segregation of proteins at these two poles (cytokines and chemokines receptors at the front side while mitochondria and integrins of the rear one) assures the functional motility of the T cell. Mitochondria relocation within the T cell is mediated by microtubules in a Ca^2+^ dependent fashion. This process is orchestrated by the MTOC (microtubules organizing center) which controls microtubules polymerization and then mitochondria localization in a Ca^2+^ -dependent fashion. The definition of T cell polarity is mandatory for a proficient T cell migration with, on one side, chemokine receptors guiding the movement at the leading edge while, on the other side, adhesion molecules controlling T cell adhesion hence providing an antithetic force. Lastly, the endocytic compartment, apart from the recycling of molecules, promotes the fine compartmentalization and the amplification of the signal with the juxtapositioning of molecules and proteins. TCR-T cell receptor; MTOC-MicroTubule Organizing Center; ATP-Adenosine TriPhosphate.

In resting conditions, T lymphocytes present a spherical shape retained by the cytoskeleton tension and, in particular, by the intermediate filaments and the cortical actin (4, 5). T lymphocyte surface is “decorated” with microvilli sustained by parallel bundles of highly dynamic actin filaments (6, 7) (**Figure 1**). These structures allow T cells to sense the surrounding microenvironment and, importantly, they promote signalling compartmentalization at their tips, leading to the coalescence of proteins and receptors involved in T cell adhesion and activation, as integrin α_4_β_7_-Very Late Antigen 4 (VLA-4), L-selectin, chemokines receptors as CXCR4 (8, 9) and T cell receptor (TCR) complexes (6, 10–14). At the tip of microvilli, proteins are found in close proximity thus prompting an easier and more efficient “scanning” of the APC surface in search of the peptide-major histocompatibility complex (pMHC) and, at the same time, increasing the avidity for subsequent interaction of the two cells. Indeed, following T cells adhesion and activation, microvilli are resorbed and integrin avidity is upregulated in a process mediated by ERMs proteins (ezrin, radixin and moesin), acting as a bridge between the PM and the actin cytoskeleton (15–17). Notably, it has been recently proposed that membrane curvature could also promote signalling compartmentalization within microvilli tip (18). This topic has been extensively discussed elsewhere (19).

In this landscape, a particular focus should be made on the actin cytoskeleton bearing a ubiquitous but fundamental role in multiple cellular processes. As for T cells, the actin cytoskeleton has a key importance in their activation, mainly during the formation and maintenance of a specialized junction named the Immunological Synapse (IS). In accordance to this, recently, it has become clear that mechanical and biochemical signals at the IS are integrated by actin dynamics (20).

Besides cytoskeleton, signalling compartmentalization in T cells is orchestrated also by “small (10–200 nm), heterogeneous, highly dynamic, sterol- and sphingolipid-enriched domains that compartmentalize cellular processes” (21, 22), defined as lipid rafts. Even if, due to technical issues, their description and existence has been debated for years, it has been clear from the beginning that their main feature is the ability to promote signalling *via* proteins juxtaposition (controlling the inclusion/exclusion of proteins) thus generating “signalling hubs”. Giving their limited size, lipid rafts can welcome only a limited number of proteins which probably stand among 10-30 proteins (22, 23). The partitioning of molecules within their structure can be regulated by multiple factors including the intrinsic molecule state, the signalling state of the cell and post-translational modifications (PTMs). Interestingly, lipid rafts are not stand alone structure by they are connected to the cytoskeleton *via* actin-binding proteins as ezrin and filamin acquiring then the definition of “floating island” or “flying kites” (21, 24).

The advancements in imaging techniques, with the introduction of single molecule and scanning confocal imaging, have overcome this primordial separation of these compartments. For instance, the “Picket and Fence Model” postulated by Kusumi and colleagues defined confinements area within the membrane (between 30 and 700nm) where transmembrane proteins are anchored and lined up along a fence of cytoskeletal proteins (25). This model arises from the evidence that transmembrane proteins can also move within confined areas and they have to “hop” when changing it. This concept was expanded by the definition of the “proteins island model” in the PM. Protein islands (both rafts and non-rafts islands) are actin-rich areas where membrane-associated proteins are clustered (26, 27) surrounded by a “sea” on protein-free regions. It has been observed that, in activated membranes, rafts and non-rafts regions presented more frequent contacts, a feature that probably shapes and influences their functionality and morphology (26). Interestingly, actin cytoskeleton is mandatory for their establishment, while cholesterol depletion does not impair proteins distribution between rafts and non-rafts regions, implying a superior organization order (26). Even if reports in this direction are still missing, it could not be excluded that protein islands may also have a role in cell-cell communication, membrane trafficking and membrane fusion.

Compelling evidence also indicates that the endocytic compartment works as a signalling hub within the cell (**Figure 1**). As elegantly revised by Scita and Di Fiore (18), endosomes sustain the signal originated by the PM and, at the same time, allow the generation of unique signals. This is possible thanks to their small volume which favors the coincidence of detectors, the specific enrichment of some lipids, the rapid microtubule-mediated transport of molecules; moreover, the endosome acidic pH might trigger and regulate specific enzymatic functions. Even if additional studies are required to model and experimentally validate endosome dynamics, it is clear how endocytosis provides a controlled spatial and temporal dimension for distinct signaling pathways.

Overall, the spatial ordering of molecular players in distinct cellular compartment enables the complexity of multiple signaling events, a feature which is mandatory for a proficient T cell migration and activation (28).

## Signalling compartmentalization in T cells

Even if in resting conditions T cells present a round-shaped morphology, they acquire functional polarity upon stimulation by a variety of ligands. This is particularly evident during T cell migration and priming, when the definition of a cellular polarity allows the maintenance of active and distinct signalling compartments. In the past, our laboratory has analyzed the mechanisms responsible for signaling amplification and compartmentalization during these two processes, focusing on the role of CD28 and chemokine receptors governed by the actin cytoskeleton. In this manuscript, we will focus on the contribution of the aforementioned players in T cell migration and activation.

### CD28 at the crossroad of cytoskeleton and lipid rafts

T cell priming starts in lymph nodes (LNs) with the formation of a stable interaction between naive T cells and antigen presenting dendritic cells (DCs). This represents a very sensitive process ultimately leading to multiple cellular responses, including T cell proliferation and the secretion of a wide range of cytokines, chemokines and cytotoxic mediators. By the use of two-photon microscopy, seminal studies unveiled the kinetics of this event *in vivo* by proposing the 3-stage paradigm: upon antigen encounter, T cells engaged first transient serial interactions (phase 1) and next stable contacts (phase 2) with antigen-loaded DCs; finally they increased their motility, detached and proliferate (phase 3) (29). In particular, the interactions between T cells and APCs are transient between 2 and 8h following the first encounters, stable between 8 and 24h, and again transient by 24–36h (30, 31).

The stability of T cell-DC interaction is determined by multiple interconnected signals, from TCRs as well as stimulatory and inhibitory receptors that are integrated in a specialized membrane junction named the “immunological synapse”-IS (32, 33) (**Figure 1**). In T lymphocytes, signalling events occurring at this platform cause multiple downstream effects ranging from the dynamic rearrangement of the actin cytoskeleton, and the initiation of a gene expression cascade ultimately leading to the generation of effector and memory T cells (34–36). At the IS, the duration of distinct molecular signals including the amplitude and kinetics of intracellular Ca^2+^ waves ranges between few minutes to hours (37).

The formation of the IS is initiated with the extension of filopodia and lamellipodia from the T cell toward the APC. The interaction of the two cells leads to the establishment of a F-actin rich interface. Then, TCR and co-stimulatory molecules, including CD28, trigger the reorganization of the cytoskeleton with the recruitment of the actin polymerization machinery and its regulatory proteins at the IS where, in a positive-feedback loop, they promote the maintenance of the TCR signalling (38, 39). Actin segregates into radial asymmetric zones defined as the supramolecular activation clusters (SMACs) (34, 40–42). We can distinguish the cSMAC (central SMAC), comprising the TCR and co-stimulation molecules; an outer ring named as pSMAC (peripheral SMAC) containing the LFA-1 (41, 43) and a distal SMAC (dSMAC) including the CD43 and CD45 (44, 45).

Mechanistically, several protein tyrosine kinases (PTKs), including Src family PTKs such as Lck and Fyn and the Syk family PTK zeta chain of TCR-associated protein 70 (ZAP-70) are brought into proximity of the CD3 complex upon TCR engagement (46). There, Lck or Fyn causes the phosphorylation of the immunoreceptor tyrosine-based activation motifs (ITAM) in the CD3 subunits. Tyrosine phosphorylation of CD3 provides the binding site for ZAP-70 *via* its SH2 domain, and then Lck or Fyn activates ZAP-70 by phosphorylation (47, 48). ZAP-70 activation in turn favored the phosphorylation of downstream adaptors, including the linker for activation of T cells (LAT) and SH2 domain-containing leukocyte phosphoprotein of 76 kDa (SLP-76) acting as scaffolds to recruit additional signalling molecules. As a consequence, multiple signalling pathways are activated at the IS eventually leading to T-cell activation, proliferation, and differentiation (49).

Importantly, in naïve T cells, the outcome of TCR stimulation is regulated by costimulatory signals. Among them, the CD28-mediated signalling strongly influences T cell priming. At the IS, CD28 signals lower T cell activation threshold by enabling an effective priming by few antigenic complexes (40, 50, 51). When CD28 is recruited at the IS, it promotes the recruitment of multiple downstream interactors at its cytoplasmic tail. Among them, the phosphoinositide 3-kinase (PI3K) (52), Lck (53, 54), growth factor receptor-bound protein 2 (Grb2) (55), Grb2-related adaptor protein (Gads) (56), IL2-inducible T cell kinase (Itk), the guaninenucleotide exchange factor Vav (57), Akt (58), protein phosphatase 2A (PP2A) (59, 60), and protein kinase C theta (PKCθ) (57). With respect to PKCθ, it has been reported that CD28-mediated signals are required for the specific localization of this kinase to the center region of the IS through its V3 motif (61).

As well, CD28 attends the selective sorting of molecular interactors in lipid membrane domains, acting as privileged sites in which signals are protected and amplified. Indeed, we showed that the CD28 co-stimulation of the TCR signaling cascade is based on lipid rafts (62). Next, we found that the kinase Lck is recruited into CD28-signaling rafts and directed to the IS upon CD28 engagement by a process requiring the CD28 COOH-terminal PxxPP motif and Vav-1, key regulator of the actin cytoskeleton rearrangements (63). Of note, IS lipid microdomains are also enriched in TCR signalling proteins, including the Src-family kinase Fyn, the adapter protein LAT, phosphoprotein associated with glycosphingolipid-enriched domains (PAG) or Csk-activating protein (Cbp) and Lck-interacting molecule (LIME) (33, 64). Interestingly, the partitioning of Lck and LAT at the IS lipid microdomains is dictated by the post-translational modifications (PTM, including protein S-acylation) (65–67). In this regard, a recent report showed that S-acylation of the plasma membrane channel ORAI1 is crucial for the selective trapping of this channel in cholesterol-rich lipid microdomains at the IS where it controls the local Ca^2+^ fluxes leading to T cell activation (68).

Furthermore, according to the protein islands theory, LAT clusters appear to aggregate with CD3/CD28 complexes in the activating surface of T cells (26). LAT acts a central mediator for T cell activation dictating, once phosphorylated, the co-clustering of CD2 and Lck in membrane discrete microdomains *via* protein-protein interactions in a process initiated by F-actin and actin-associated proteins. Beside this, LAT also regulate calcium dynamics at the IS and Ras signalling (28). As mentioned before, CD28 acts as a master regulator of actin cytoskeleton rearrangements during T cell activation by tuning the actin polymerization machinery. This process is under the control of several interactors: upon TCR-engagement, the kynase ZAP-70 phosphorylates the adaptors SLP-76 that then binds Nck and the guanine nucleotide exchange factor Vav-1. More, Nck constitutively associated with WASp (69, 70) thus acting as a bridge to recruit WASp itself to the SLP-76 signaling complex. In association with SLP-76, Vav-1 mediates the exchange of GDP- to GTP-bound Cdc42, Rho family GTPases that interacts with the conserved VCA domain of WASp allowing its binding to the Arp2/3 complex. Once bound to the VCA domain, Arp2/3 promotes the branching of the actin polymerization and rearrangement at the T cell-APC contact site (71). Arp2/3 cooperates with filamins that are actin crosslinking proteins. In this landscape of interactors, we pointed out the actin-binding protein Filamin-A (FLNa) as the molecular partner of CD28 both in the reshaping of the actin cytoskeleton and in the lipid rafts recruitment at the IS (72). In this study, we showed that the COOH-terminal PxxPP motif of CD28 is required for CD28–FLNa association, and that FLNa has a direct role in CD28 signalling by recruiting Cdc42 at the site of Vav-1 activation. Vav-1 plays a crucial role in the regulation of the CD28 costimulation. Indeed, it has been shown that the adaptor molecule Cbl-b controls the CD28 dependence of T-cell activation by selectively suppressing TCR-mediated Vav activation (73). Cytoskeletal actin dynamics are also regulated by the phosphatidylinositol bisphosphate (PIP2) produced by the activity of the PIP5K enzymes. In this regard, we and other showed that, in collaboration with PIP5Kα and Vav1, PIP5Kβ promotes actin polymerization and CD28 signaling in human T cells (74, 75). Other reports further support the relevance of the dynamic regulation of actin in CD28-mediated costimulation by linking the actin-uncapping proteins Rltpr (76) and CapZIP (77) to the CD28 costimulatory signalling.

More recently, a phenomenological agent-based model has been developed for assessing the contribution of actin-driven forces to IS formation and CD28 localization. By applying this model, authors proposed that although CD28 can reach the IS center by passively following TCR clusters, the ring-like pattern of CD28 at the synapse is determined by the coupling to the actin cytoskeleton (78).

Taken together this evidence endorses the outstanding role of CD28 as a signalling hub in T cells finely tuning cytoskeletal dynamics and lipid rafts reorganization.

Beside CD28, which positively regulates T cell activation, other inhibitory molecules are present on the T cell surface. Among these, the most characterized are CTLA4 and PD1, whose importance rapidly increased in recent years as targets for immune-mediated therapies. These are recruited within the cSMAC together with their downstream mediators and here they compete with CD28 ligands (B7-1/CD80 and B7-2/CD86) for binding, thus promoting the establishment of T cell anergy (5, 34, 79). Interestingly, most of CTLA4 seems to reside within endocytic vesicles, a mechanism facilitating its signalling with a fine compartmentalization (5, 79). Similarly to CTLA4, also PD-1 presents a minimal expression in resting conditions, further increased after T cell activation (79). Thanks to the binding to PD-L1 and PD-L2, it abolishes IL-2 production in T cells and, albeit only in some settings, it also induce T cells apoptosis (79). As was recently revised, both these molecules affect T cell motility reducing its ability to “pause” when encountering the cognate APC thus raising the threshold for IS formation in the “reverse-stop signal model” (80, 81). This effect seems to be mediated by phosphatidylinositol 3-kinase, Vav-1, Cdc42, and myosin light chain MLC kinase (82) which also affect T cell motility to inflamed sites (80). In addition, in was reported that PD-1 mediates the inhibition of T cell function acting mostly on CD28 rather than on TCR (83).

### Chemokine receptors

In T cells, the activation of chemokine receptor signalling contributes to the spatial and temporal repositioning ofwfi 2 intracellular and membrane-bound players, ultimately defining T cell polarity. During migration, polarity refers to the ability of cells to change their morphology in response to chemoattractants, and to maintain a stable asymmetric shape with two poles: the leading edge, which protrudes at the cell front, and the rear edge (termed uropod in leukocytes), at the back (84). This process, which is initiated by chemokine receptor signalling and adhesive interactions with the extracellular matrix (ECM), increases the sensitivity toward chemokine gradients, by the selective recruitment of chemokine receptors at the T cell front (85) (**Figure 1**). Compartmentalization of the PM into distinct lipid microdomains is pivotal in establishing and maintaining leukocyte polarity and perturbation of lipid microdomains inhibits both cell polarization and migration (85, 86).

The spatial organization of chemokine receptors into dimers and higher-ordered oligomers further adds to the complexity of possible GPCR arrangements, and consequently modulation of signaling (87). Recent studies shed light on how cholesterol dictates the spatial organization of GPCRs within the PM. in particular, it has been proposed that cholesterol promotes the oligomerization of chemokine receptors at the PM that ultimately enabling the integration of distinct signaling pathways at the receptor-membrane interface (88). Previously, it has been shown that the CXCR4 and CCR5 receptors associate to GM3-enriched lipid rafts and are consequently redistributed to the leading edge of moving cells. Interestingly, both CXCR4 and CCR5 directly interact with FLNa, that actively modulates their signalling pathways. Indeed, the specific blockade of CXCR4–FLNa interaction inhibited CXCL12-induced chemotaxis in T cells. As for CXCR4, filamin-A expression did not affect CCR5-mediated Ca^2+^ flux, but regulated F-actin remodelling (89).

Chemokine receptors play a pivotal role during T cell activation, too. Long-lasting interactions between T cells and APCs are dependent on antigens (90, 91), but antigen-specific interactions are preceded by antigen-independent, chemokine-promoted adhesive contacts in the T cell-APC pair, enabling T cells to scan the surface of their cellular partners (92–94). Although the induction of cell polarity at the IS was thought to be dependent on TCR triggering, we have shown that CXCR4-induced activation of LFA-1 at the contact site with APCs starts MTOC and mitochondria relocation towards the upcoming IS (95). Importantly, we found that, by recruiting mitochondria to the IS, LFA-1 sustains and amplifies the upcoming TCR-induced Ca^2+^ signalling, indicating that establishment of T-cell polarity is pivotal to a prompt and sustained T cell activation (95) (**Figure 1**). Interestingly, by bringing mitochondria and ORAI channels into close proximity and by re-organizing plasma membrane calcium ATPases (PMCAs) into discrete regions co-localizing with mitochondria, the IS prevents Ca^2+^ -dependent channel inactivation and reduce local Ca^2+^-dependent PMCA modulation (96).

The tight spatial and temporal regulation of cytosolic calcium (Ca^2+^) is of paramount importance for multiple T cell effector functions as differentiation, proliferation, metabolism, cytokine release and cytotoxicity. The IS indeed controls Ca^2+^ microdomains by bringing mitochondria and ORAI channels into close proximity and favoring the segregation of PMCA into distinct PM domains. The proteins and organelles re-distribution allows mitochondria to rapidly take up the inflowing Ca^2+^, thereby avoiding high Ca^2+^ microdomains close to ORAI channels, which prevents Ca^2+^-dependent channel inactivation and reduce local Ca^2+^-dependent PMCA modulation. This optimizes net Ca^2+^ influx at the IS (96).

The mechanisms responsible for Ca^2+^ signal compartmentalization in T cells have been extensively explored and described (97). Early recruitment of mitochondria at the T-cell IS occurs independently of TCR stimulation and through a mechanism requiring chemokine receptor signalling (95). Interestingly, we had also shown that chemokine receptor signaling induces accumulation of mitochondria at the uropode of migrating cells, where they are required to sustain phosphorylation of the MLC, a key step in high-speed moving cells (98).

In addition to shaping T cells for effective signaling, chemokine receptors directly support the IS stabilization and indeed and T cell activation. We had demonstrated that CXCR4 and CCR5 are stably recruited into the IS by APC-secreted chemokines (70). In this context, chemokine receptors contribute to the amplification of the TCR signalling acting as powerful costimulatory molecules (99). Indeed, their recruitment at the IS prolong the duration of the T cell–APC interaction and strengthen T cell–APC pair attraction ultimately avoiding premature splitting due to chemoattractant sources (99).

Of note, TCR engagement significantly impacts on chemokine receptor signaling properties by favoring the selective triggering of distinct downstream players (79). Canonically, chemokine signaling, initiated following ligand binding, causes the dissociation of the Gai and Gbg subunits of the heterotrimeric G proteins, leading to calcium flux, PI3K triggering and the activation of the small Rho GTPases signaling. However, alternative signalling pathways resulting from the coupling with other G proteins have also been reported for these receptors (100). Importantly, we showed that at the IS chemokines promote the preferential association of the receptor CCR5 with the Gq/11 subunit instead of Gi one (99).

The functional versatility of chemokine receptors in the context of T cell activation may depend on their ability to heterodimerize with other GPCRs. For example, we showed that CXCR4/CCR5-mediated costimulation grounded on their ability to form heterodimers at the IS (101).

In addition, inhibitory molecules (as CTLA4) have been demonstrated to alter the motility both *via* the up-regulation of chemokine receptors (CCR5 and CCR7) and by the increase in the sensitivity to their respective chemokines (CCL4 (MIP-1β), CXCL12 (SDF1α) and CCL19). This evidence leads to the proposal of a model for chemotaxis integrating CD28 and CTLA-4 signals *via* the G protein-coupled receptor kinase GRK. CD28 triggers CCR5 phosphorylation *via* GRK, while CTLA-4 engagement inactivates GRK2 counteracting this mechanism (80).

More recently, an additional mechanism elucidating CXCL12-induced T cell co-stimulation has been proposed. Smith and colleagues showed that the chemokine enhances the number, stability, and phosphorylation of SLP-76 microclusters formed in response to stimulation of the TCR. This results in proximity of SLP-76 and ZAP-70 clusters and in enhanced TCR-dependent gene expression (102).

Multiple studies worked to clarify whether other chemokines preferentially act as co-stimulatory partners for the TCR ultimately promoting T-cell activation. Recently, it has been proposed that CCR7, which drives T cell and DC migration and trafficking in LNs, colocalizes with the TCR at the IS, within sub-synaptic vesicles. There, CCR7 promotes and prolongs ZAP70 activity, resulting in T cell costimulation (103).

All these data, together with many more that we could not include in our discussion, suggest that T cell priming results from a timely and spatially regulated interplay between adhesive and chemoattractant forces mainly occurring in LNs, enabling T cell scanning for the cognate antigen and the formation of long-lasting interaction upon recognition (104).

## Congenital defects in cytoskeletal proteins lead to impairment of T cell activation

Perturbations in the equilibrium between adhesive and chemotactic forces leads to defects in the formation of a productive IS, due to the instability of the T cell-APC mating (105). Of note, different inborn errors in genes encoding for proteins controlling these functions, lead to syndromes linked to defects in T cell motility and/or activation (106).

The Warts, Hypogammaglobulinemia, Infections, and Myelokathexis (WHIM) syndrome is a primary immunodeficiency disorder in which a genetic mutation impairs CXCR4 internalization and enhances its responsiveness to CXCL12. WHIM patients experience a wide range of symptoms, including recurring infections, human papillomavirus (HPV)-induced warts, reduced long-term immunoglobulin G (IgG) titers, myelokathexis, and leukopenia (107). The dominant mutations in the chemokine receptor CXCR4 lead to the truncation of its carboxy-terminal domain, ultimately resulting in a defective ability of the receptor to internalize after binding its ligand. As a consequence, immune cells bearing the WHIM-mutant receptor display increased signalling and enhanced migration in response to chemokine stimulation (108). We observed that, in contrast to the wild-type CXCR4, the WHIM-mutant CXCR4 failed to be recruited into the IS and impaired the formation of long-lasting T-APC interactions, thus limiting T cell priming and immune responses to antigens (109). Thus, the hyperfunctional WHIM-mutant CXCR4 favors motility over formation of stable IS, resulting in aberrant T cell activation (109).

The Wiskott-Aldrich syndrome (WAS) is a primary immunodeficiency determined by mutations in the WAS-protein (WASp), a member of a larger family of proteins (WASP family) that functions as nucleation-promoting factors for the Arp2/3 complex, which drives the generation of branched actin filaments (110). WASp is exclusively expressed in cells of the haematopoietic lineage and its loss-of-function mutations cause a syndrome characterized by a broad range of clinical signs, with patients showing an increased susceptibility to infections, haemorrhages, eczema and different autoimmune disorders (111). Upon TCR engagement, WASP is recruited to the IS where it interacts with VAV, RAC and Cdc42 and is activated by VAV effectors (6, 112, 113). WAS patients present alterations in T cell actin cytoskeleton dynamics (114, 115). WASp^-/-^ T cells fail to polymerize actin in response to anti-CD3 stimulation, and show defective IS. The disorganized signaling platforms of WASp^-/-^ T cells do not allow complete and efficient cellular activation and, consequently, T cells from WAS patients show decreased cell proliferation and cell survival (111). Interestingly, this is linked with a severe impairment in CD28 internalization possibly caused by the formation of the functional complex WASp/SNX9/p85/CD28 (116).

Mutations in the WASp-interacting protein (WIP) can also determine a syndrome with clinical signs similar to WAS. WIP is involved in the regulation of WASp activity by promoting its stability, activation and localization to sites of active actin polymerization. Moreover, independently from WASp, WIP regulates actin cytoskeleton in lymphocytes affecting the homing of T cells to infected tissues (117).

Additional immunodeficiencies caused by defects in actin-binding proteins and leading to T cell synapse instability have been described. Among them, the deficiency of the ARPC1B protein, part of the Arp2/3 complex, caused the emission of aberrant actin-rich structures, including spikes and long filopodia-like structures, both in the context of 2D IS and contact with APC (118). Thus, patients suffering of ARPC1B deficiency show defects in T cell proliferation and cytotoxic activity. Interestingly, ARPC1B also contributes to the recycling of the TCR, CD8 and GLUT1 (119), thus causing reduced expression of these molecules in ARPC1B-deficient CD8^+^ T cells. In addition, as a result of an impaired endosome-to-membrane recycling processes caused by a deficient actin remodeling, T cells lacking the Arp2/3 activator WASH also fail to maintain surface levels of the TCR, CD28, LFA-1 and GLUT1 molecules (120).

Although relevant for T cell activity, other defects, including HEM1 and WDR1 deficiencies, might not be solely explained by defective IS and have been reviewed elsewhere (121). Further investigations are needed to mechanistically explore the role of CD28 and other costimulatory molecules in these disorders.

## Future directions

Although here we focused our discussion on chemical signaling, it must be noted that mechanical signals control T cell functions and are required for cell polarization, migration and activation. In particular, membrane curvature seems to initiate signaling events resulting in the organization of larger signaling platforms (122, 123). In both neutrophils and CD8+ T cells, cell polarization was shown to be dependent on local increase of plasma membrane curvature induced by initial adhesion (122). The curved membrane can orchestrate the formation of signaling platforms through the Bin-Amphiphysin-Rvs (BAR) superfamily. BAR proteins induce, regulate and detect membrane curvature (124) and recruit to the curved membrane other proteins, including regulators of actin dynamics.

While the N-BAR and the F-BAR proteins are generally associated with membrane invaginations, the I-BAR are present in various membrane protrusions (125) and involved in microvilli formation (126). Little is known about the role of BAR proteins in T cell functions. The I-BAR IRSp53 is expressed in T cells and essential for the release of HIV particles through a pathway involving Rac1, Wave2 and Arp2/3 (127, 128), but its role in microvilli formation and TCR signaling is unknown. On the other hand, sorting nexin 9 (SNX9), which belongs to the N-BAR subfamily but regulates filopodia formation (129), forms a signaling complex on endocytic vesicles with CD28, WASp and p85 in T cells triggered by CD3/CD28 antibodies (116). In a feed-forward fashion, SNX9 itself was recently shown, once recruited to the IS, to generate membrane tubulation out of CD28 clusters with these dynamic structures regulating both CD28 phosphorylation status and IL-2 production (130).

Further studies will be required to shed light on the role of BAR domain proteins and the membrane curvature in signaling compartmentalization and T cell functions. However, it seems conceivable that cells employ a combination of physical and biochemical forces to tune the formation of structures and domains on the plasma membrane (131). How the integration of the different forces occurred in T cells will be an interesting subject for future investigations.

In addition, accumulating evidence suggests that mechanical forces are key determinants in initiating signaling through the TCR that clearly acts as a membrane mechanoreceptor. In this regard, very recently a new model for TCR triggering has been proposed (132). Indeed, the TCR Bending Mechanosignal (TBM) model predicts that mechanical forces might cause membrane curvature around engaged pMHC/TCR complexes; such mechanical cue is necessary to reach the energy threshold required for the triggering of the signalling cascade ultimately activating T responses (132).

Of note, the investigation of whether and how mechanical signals control costimulatory molecules, as CD28 and chemokine receptors, would be an interesting advancement in this field.

Signalling compartmentalization is essential for immune cells to respond with high specificity and sensitivity. Thus, achieving a deeper understanding of the mechanisms regulating the generation of signalling compartments during T cell migration and activation will be important to modulate immune responses with future therapeutics and will be vital to design effective CAR-T cells.

## Author contributions

BM, CL, AV conceived and wrote the manuscript. CL drew the figure. All authors contributed to the article and approved the submitted version.

## Funding

BM and AV received research grant from Istituto di Ricerca pediatrica Fondazione Città della Speranza

## Conflict of interest

The authors declare that the research was conducted in the absence of any commercial or financial relationships that could be construed as a potential conflict of interest.

## Publisher’s note

All claims expressed in this article are solely those of the authors and do not necessarily represent those of their affiliated organizations, or those of the publisher, the editors and the reviewers. Any product that may be evaluated in this article, or claim that may be made by its manufacturer, is not guaranteedor endorsed by the publisher.
